# Automating incidence and prevalence analysis in open cohorts

**DOI:** 10.1186/s12874-024-02266-7

**Published:** 2024-07-04

**Authors:** Neil Cockburn, Ben Hammond, Illin Gani, Samuel Cusworth, Aditya Acharya, Krishna Gokhale, Rasiah Thayakaran, Francesca Crowe, Sonica Minhas, William Parry Smith, Beck Taylor, Krishnarajah Nirantharakumar, Joht Singh Chandan

**Affiliations:** 1 Institute of Applied Health Research, University of Birmingham, Birmingham, West Midlands UK; 2 NIHR Blood and Transplant Research Unit (BTRU) in Precision Transplant and Cellular Therapeutics, University of Birmingham, Birmingham, UK; 3 Department of Obstetrics and Gynaecology, Shrewsbury and Telford Hospitals NHS Trust, Telford, Shropshire UK; 4 Keele University, Keele, Staffordshire UK; 5 Warwick Medical School, Warwick University, Coventry, Warwickshire UK

## Abstract

**Motivation:**

Data is increasingly used for improvement and research in public health, especially administrative data such as that collected in electronic health records. Patients enter and exit these typically open-cohort datasets non-uniformly; this can render simple questions about incidence and prevalence time-consuming and with unnecessary variation between analyses. We therefore developed methods to automate analysis of incidence and prevalence in open cohort datasets, to improve transparency, productivity and reproducibility of analyses.

**Implementation:**

We provide both a code-free set of rules for incidence and prevalence that can be applied to any open cohort, and a python Command Line Interface implementation of these rules requiring python 3.9 or later.

**General features:**

The Command Line Interface is used to calculate incidence and point prevalence time series from open cohort data. The ruleset can be used in developing other implementations or can be rearranged to form other analytical questions such as period prevalence.

**Availability:**

The command line interface is freely available from https://github.com/THINKINGGroup/analogy_publication.

**Supplementary Information:**

The online version contains supplementary material available at 10.1186/s12874-024-02266-7.

## Motivation

### Introduction

With the growing demand for and accessibility of administrative healthcare databases, analysis of the large datasets available require scalable analysis methods and dissemination [1]. Solutions must be readily deployable, reliably reproducible, minimise additional resource or capabilities requirements, and adhere to open science and code principles [2]. However, such data analysis solutions require domain knowledge, technical skills and significant time investment [3] and so few generalisable solutions have been deployed [4]. Our team have previously developed the ‘Data Extractor for Epidemiological Research’ (DExtER) [5] to automate analysis-ready extraction from healthcare data according to specific epidemiological study designs, and in this paper outline open analytics to handle analysis-ready data outputs.

### Open code

Open Code refers to making research code available as a research output, for example by creating open source software or sharing electronic notebooks. By making research reproducible, replicable and transparent, this approach promotes productivity, innovation and trust in science [6, 7]. However, its adoption in current research can be limited by concerns about personal data privacy issues, resource constraints and intellectual property [8]. In the context of healthcare data analysis and epidemiological research, adopting open code principles can increase the impact of tools and research outputs. For example, openPrescribing [9] is a tool to improve the quality, safety, and cost-effectiveness of prescribing practices and provides open access to all code and analysis via GitHub. Open source projects allow for greater scrutiny, adaptability, and trust, and can address major challenges in healthcare research [10].

### Rationale for automation

Automating health data analysis enables greater validity and attention to methods by standardising processes and analyses, and increase productivity of analytic resources [11]. Incidence and prevalence code is often generated per-analysis, which reduces standardisation of the analysis and leads to issues in reproducibility and comparability. Other work has previously noted the impact that choices in calculating incidence and prevalence can have on analytical results [12], and Ostropolets et al. showed that analysis choices in parameters such as incidence definition, age, and data source can generate 1000-fold differences in incidence rate estimates [13]. This could allow analysts to focus more time on implications and critical analysis of their findings, leading to more valuable insights and a greater understanding of population health. With the growing quantity of healthcare data increasing year on year, due to advances in healthcare technology, population growth, and an ageing population, automated analysis will be essential to using this data to its full potential.

### Open cohorts, incidence, and prevalence

Open cohorts generate datasets where participants can continuously enter and exit the cohort throughout the study period of the cohort [14]; this results in non-uniform follow up periods during which events can be observed, and adds complexity to analysis. Incidence and prevalence are used extensively in epidemiology to describe the population health needs, for example using data from open cohorts, and are used by policy makers to identify and plan for disease-associated burden of disease by developing health services, research programmes or preventative policies [15, 16]. Prevalence, “*the proportion of a population who have specific characteristics in a given time period*”, informs the need for health and social care services, while incidence, “*the number of new cases of a characteristic that develop in a population in a specified time period*”, is crucial in tracking disease causes, trends and evaluating the effectiveness of interventions [17]. Reducing unwarranted variation is urgently necessary and automated incidence and prevalence can provide efficient and reproducible methods across systems, datasets, and populations. Automated analysis can thus support a more precise understanding of disease patterns across times, places, and populations, helping to identify health inequalities and inform population health needs interventions [18]. This enables data-driven decision-making in public health, and can contribute to the overall improvement of health services and equity.

## Implementation

In this paper we provide both an explicit implementation-free set of rules for incidence and prevalence calculation that can be applied to any open cohort, and a command line interface for a python implementation of these rules.

### Analysis approach

We provide methods for calculating point prevalence and period incidence time series, and calculate confidence intervals using Byar’s method as described elsewhere [19]. Other types of incidence and prevalence metrics can be calculated by rearranging the rules provided; for example, period prevalence can be calculated using the denominator rules from incidence and the incidence from prevalence. Point prevalence is the proportion of a population with a characteristic such as a diagnosis at a given point in time (e.g. proportion with a diagnosis of high blood pressure). Incidence is the rate at which a population experiences an event such as receiving a diagnosis over a given period of time (e.g. the rate of heart attacks).

### Data requirements

Calculating estimates from an open cohort requires that for each observation, the time at risk is calculated. Therefore, each observation must have the following information:

- **START DATE** Date on which an individual’s study participation starts.
- **END DATE** Date on which an individual’s study participation ends.
- **EVENT DATE** Date on which event occurred, or NA if not recorded.
- **PERIOD START** Date on which a point prevalence is calculated, or observation for an incidence calculation begins.
- **PERIOD END** Date on which observation for an incidence calculation ends.

### Rules

#### Point prevalence

##### Definition of numerator in point prevalence for a given population P:

$$\begin{aligned} \text {Numerator} (P) = \sum I_{n} \end{aligned}$$

where $I_{n}$ is an indicator function defined as

1 $$\begin{aligned} I_{n} = \left\{ \begin{array}{ll} 1, &{} \text {if}\ Z_n == TRUE\\ 0, &{} \text {otherwise} \end{array}\right. \end{aligned}$$

where $Z_{n}$ is True if all three statements below are True:

1. **START DATE** $\varvec{<=}$ **PERIOD START** (patient follow-up began before or on the start date for the analysis.)
2. **END DATE** $\varvec{>=}$ **PERIOD START** (The patient follow-up end date occurred on or after the start date for the analysis.)
3. **EVENT DATE** $\varvec{<=}$ **PERIOD START** (The event date occurred before or on the start date for the analysis.)

#### Definition of denominator in point prevalence for a given population *P*

 

$$\begin{aligned} \text {Denominator} (P) = \sum I_{d} \end{aligned}$$

where $I_{d}$ is an indicator function defined as

2 $$\begin{aligned} I_{d} = \left\{ \begin{array}{ll} 1, &{} \text {if}\ Z_d == TRUE\\ 0, &{} \text {otherwise} \end{array}\right. \end{aligned}$$

where $Z_{d}$ is True if both statements below are True:

1. **START DATE** $\varvec{<=}$ **PERIOD START** (patient follow-up began before or on the start date for the analysis.)
2. **END DATE** $\varvec{>=}$ **PERIOD START** (patient follow-up end date occurred on or after the start date for the analysis.)

#### Period incidence

##### Definition of numerator in incidence rate for a given population *P*:

$$\begin{aligned} \text {Numerator} (P) = \sum I_{n} \end{aligned}$$

where $I_{n}$ is an indicator function defined as

3 $$\begin{aligned} I_{n} = \left\{ \begin{array}{ll} 1, &{} \text {if}\ Z_n == TRUE\\ 0, &{} \text {otherwise} \end{array}\right. \end{aligned}$$

where $Z_{n}$ is True if all statements below are True:

1. **PERIOD START** $\varvec{<=}$ **EVENT DATE** $\varvec{<}$ **PERIOD END** (The event date occurred on or after the start date but before the end date of the analysis.)
2. **END DATE** $\varvec{>=}$ **PERIOD START** (The patient follow-up end date occurred on or after the start date for the analysis.)
3. **START DATE** $\varvec{<}$ **PERIOD END** (Patient follow-up started before the end date of the analysis.)

##### Definition of denominator in incidence rate for a given population *P*:

$$\begin{aligned} \text {Denominator} (P) = \sum PT_{d} \end{aligned}$$

where $PT_{d}$ is total person time contributed by each patient in the period of interest.

4 $$\begin{aligned} PT_{d} = \left\{ \begin{array}{ll} (\text {END\_OBSERVATION}-\text {START\_OBSERVATION})/365.25, &{} \text {if}\ Z_d == TRUE\\ 0, &{} \text {otherwise} \end{array}\right. \end{aligned}$$

where,

- **START OBSERVATION** $=$ maximum(**START DATE**, **PERIOD START**)
- **END OBSERVATION** $=$ minimum(**END DATE**, **EVENT DATE**, **PERIOD END**)

and, $Z_{d}$ is True if all statements below are True:

1. **START DATE** $\varvec{<}$ **PERIOD END** (Patient follow-up occurred before the end date of the analysis.)
2. **END DATE** $\varvec{>=}$ **PERIOD START** (The patient end date occurred on or after the start date of the analysis.)
3. **EVENT DATE** $\varvec{>=}$ **PERIOD START** or **EVENT DATE** $==$ **NA** (The event date occurred on or after the start date for the analysis.)
4. **EVENT DATE** $\varvec{>}$ **START DATE** or **EVENT DATE** $==$ **NA** (The event date occurred after patient follow up began or there was no event.)

### Python implementation

These rules have been implemented into a python command line interface (CLI) available from https://github.com/THINKINGGroup/analogy_publication and used as part of our workflow for analysis of primary care records. The CLI requires python 3.9 or above and contains example data to test. We recommend using Anaconda for open source python distribution. Below we present an example output analysing incidence and prevalence of ectopic pregnancy.

## Use case

### Ectopic pregnancy

Ectopic pregnancy presents a key risk to maternal health, and ruptured ectopic pregnancy is a predominant cause of mortality in the first trimester [20]. UK incidence and prevalence has not been reported in the literature since 2011 [21] and no study has reported the burden of disease in key subgroups. We provide the incidence and prevalence of ectopic pregnancy in the CPRD Aurum database, derived from UK primary care data.

### Study design

We extracted data for women aged 12-60 from CPRD Aurum between 2006 and 2021 [22]. 10,248,694 women were eligible for inclusion and ectopic pregnancy definitions are available in S1 & S2 Codelists. Incidence and prevalence were calculated according to Implementation.

### Overall Incidence and Prevalence

**Fig. 1:**
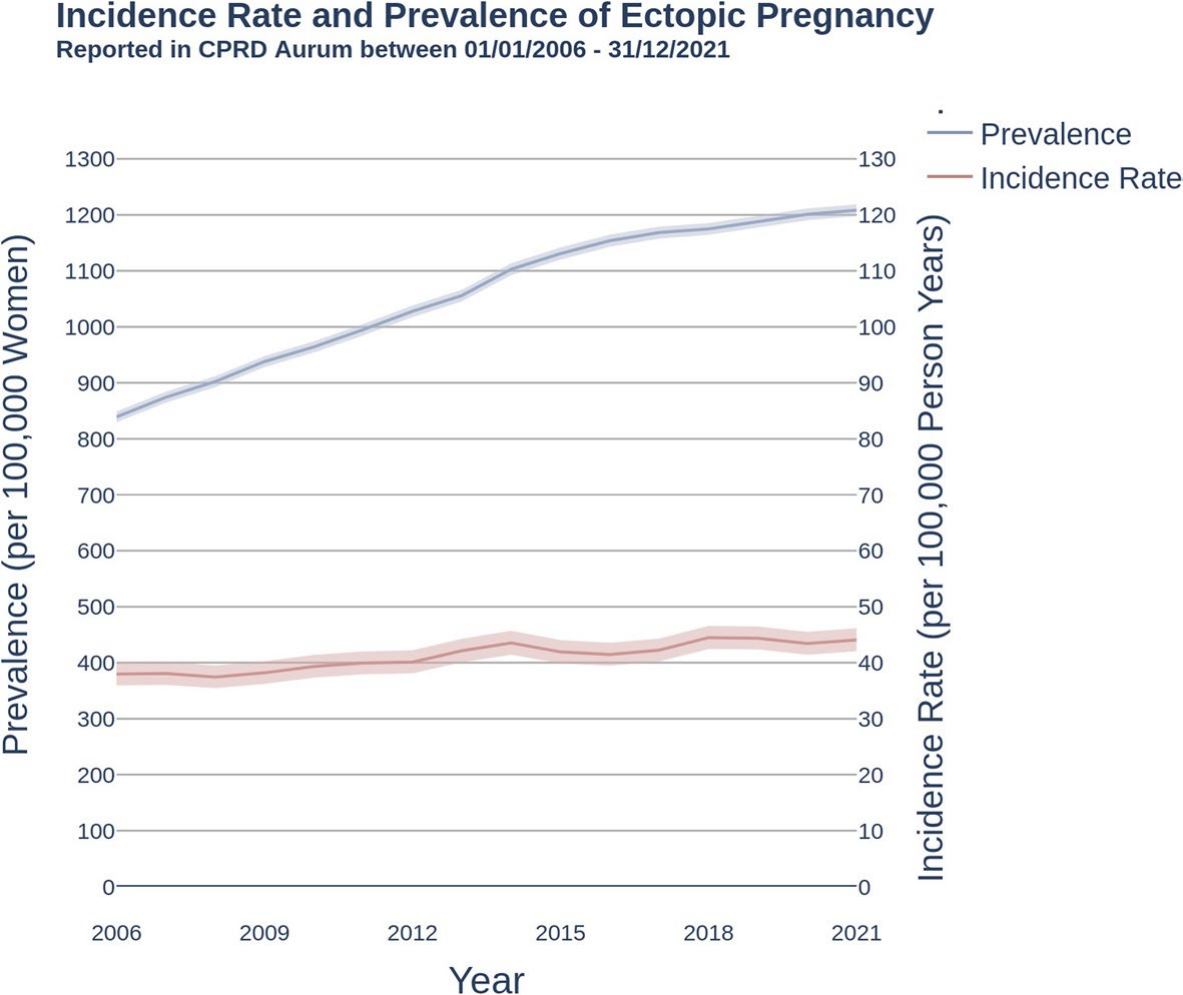
Incidence rate and lifetime prevalence of ectopic pregnancy per 100,000 women. Women aged 12-60 years of age; Clinical Practice Research Datalink Aurum; UK, 2006-2021

Figure 1 shows the incidence of ectopic pregnancy in women aged 12-60 years rose from 38.0 (95% CI: 36.0, 40.1) per 100,000 person years in 2006 to 44.1 (95% CI: 42.0, 46.2) per 100,000 person years in 2021. In the same period, the prevalence of women who had ever had a recorded diagnosis of ectopic pregnancy rose from 839.3 (95% CI: 829.7, 848.9) per 100,000 population in 2006, to reach 1209.2 per 100,000 population in 2021 (95% CI: 1197.8, 1219.1).

### Incidence and prevalence by subgroup

**Fig. 2:**
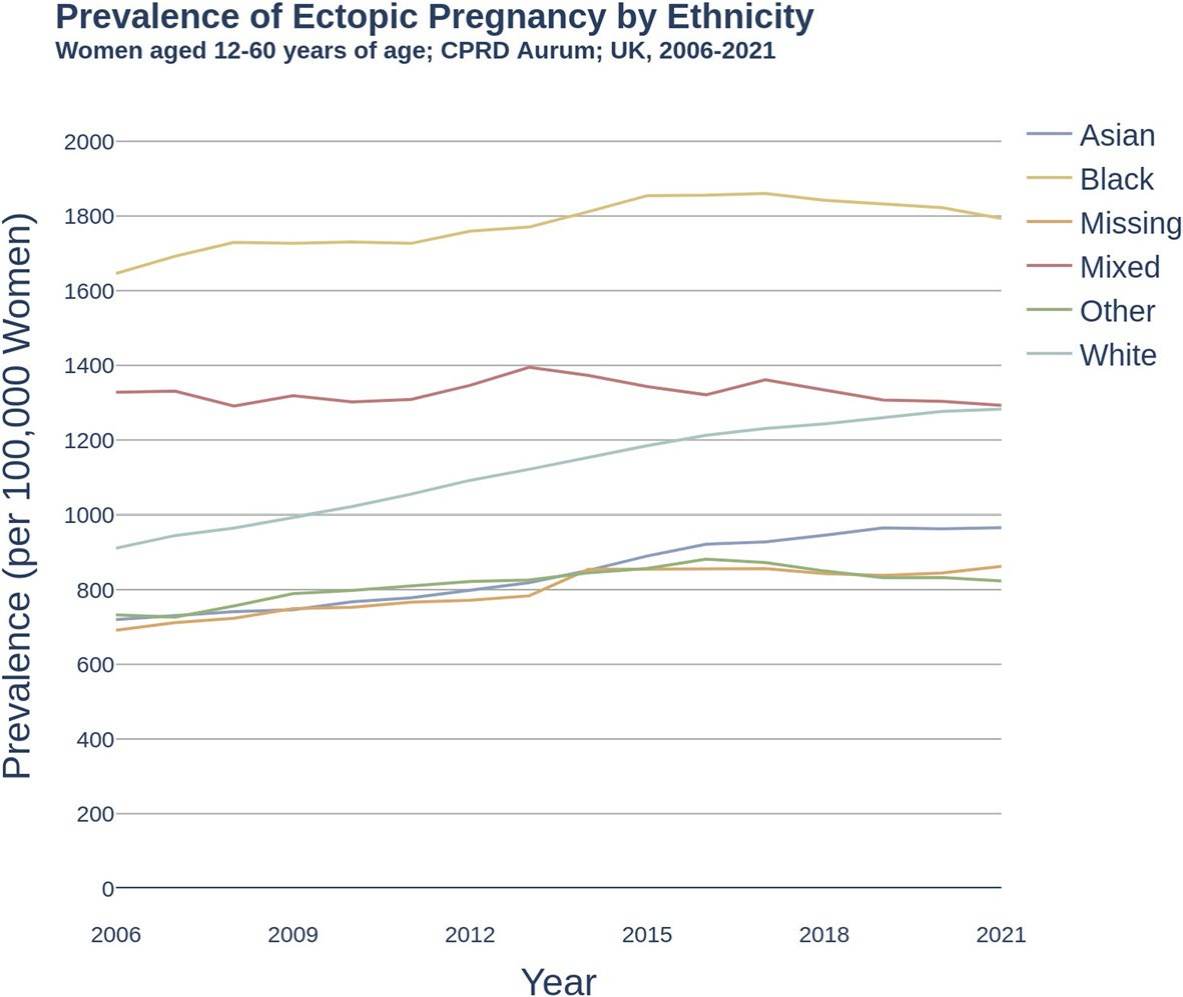
Prevalence of ectopic pregnancy per 100,000 women by ethnicity. Women aged 12-60 years of age; Clinical Practice Research Datalink Aurum; UK, 2006-2021

Between 2006-2021, ectopic pregnancy was higher in women of black ethnicity compared to white ethnicity, while no difference was observed between mixed and white ethnicity, as shown in Fig. 2 (black: 1793.5 [95% CI: 1735.0, 1853.4], mixed: 1292.8 [1215.9, 1373.3], white: 1282.4 [1269.1, 1295.8]). Women of asian, other, and missing ethnicities reported lower prevalence of ectopic pregnancy when compared to patients of white ethnicity (asian: 966.3 [95% CI: 936.1, 997.2], other: 823.6 [757.6, 893.9], missing: 862.7 [839.2, 886.7]). Additional data including deprivation and regional subgroups are reported in S3,S4 and S5 Additional Data.

## Discussion

We have demonstrated a standardised process for calculating incidence and prevalence in an open cohort dataset. Describing the underlying rules allows other analysts to reuse the rules in their own analyses and reinterpret them for other contexts such as different data sources, and aids communication and scrutiny of the analysis undertaken. We encourage readers to apply our easy-to-use CLI on their own datasets to test their analysis for replicability, and report differences to increase transparency around the effect of analysis methods, which have been shown to create substantial differences in estimates of effect sizes [23]. No UK study of the burden of ectopic pregnancy has been undertaken since 2011. However, our analysis of ectopic pregnancy is similar in design to the automated analysis estimate from PrevalenceUK, who report an annual incidence of ectopic pregnancy per woman of 46.5 per 100,000 for the UK in 2019, 4.7% larger than our estimate of 44.4 per 100,000 [24]. Three differences in analysis may explain the magnitude of difference; we used CPRD aurum only, while PrevalenceUK use a combined CPRD Aurum-Gold database; we restricted age of women included in the study to 12-60 years while PrevalenceUK likely used a whole population denominator; and differences in implementation of incidence.

### Strengths and limitations

We chose ectopic pregnancy as a use case to demonstrate the ability to rapidly identify and address gaps in research using transparent methods. However, it also reveals challenges to this automated process in specialist conditions; a more natural denominator for ectopic pregnancy might be pregnancy, rather than women of child bearing age. Biases in the source data must also be considered and studies are likely to require input from analysts or other experienced data users. For example, the 44% rise in ectopic pregnancy prevalence, concurrent with modest change in incidence, reflects better recording over time as electronic health records mature. Automated analytics in open cohorts are therefore likely to remain a specialist tool.

### Applications and future developments

We have implemented standardisation of incidence and prevalence locally, using the DExtER platform to produce a complete incidence and prevalence pipeline of analysis, and are developing tools to automate open cohort analysis using propensity score matching, cox regression, and statistical process control. Our tool’s automation and subgrouping features may have particular applications into inequalities policy making and research, by allowing rapid investigation of multiple conditions, in multiple contexts, affecting different groups of people.

## Conclusion

Many teams use common datasets such as CPRD Gold and Aurum, and exact replication of results by other teams should be feasible. Our ruleset enables analysts to use clearly defined criteria for calculating estimates, and our CLI tool can automate these calculations, for example to support sensitivity checks of results using alternative code. However barriers still exist to achieving these open science goals.

### Supplementary Information

Supplementary Material 1: S1. Ectopic Pregnancy Incident Phenome. SNOMED-CT codes used to identify ectopic pregnancy events.

Supplementary Material 2: S2. Ectopic Pregnancy Prevalent Phenome. SNOMED-CT codes used to identify ectopic pregnancy history.

Supplementary Material 3: S3. Baseline Characteristics. This table summarises characteristics of eligible women on entry to the ectopic pregnancy cohort extracted from CPRD Aurum database.

Supplementary Material 4: S4. Incidence of ectopic pregnancy overall and by subgroup. Time series of incidence of ectopic pregnancy in CPRD Aurum from 2006-2021, overall and by subgroups of Index of Multiple Deprivation, region, ethnicity and age category.

Supplementary Material 5: S5. Prevalence of ectopic pregnancy overall and by subgroup. Time series of prevalence of ectopic pregnancy in CPRD Aurum from 2006-2021, overall and by subgroups of Index of Multiple Deprivation, region, ethnicity and age category.

## Data Availability

Individual patient data are available from CPRD with valid license; all analysis results are presented in S3-5: Additional Data.
